# The Pro-Health Benefits of Morusin Administration—An Update Review

**DOI:** 10.3390/nu13093043

**Published:** 2021-08-30

**Authors:** Anita Panek-Krzyśko, Monika Stompor-Gorący

**Affiliations:** Department of Human Pathophysiology, Institute of Medical Sciences, University of Rzeszów, Warzywna 1a, 35-310 Rzeszów, Poland; anita.panek@onet.pl

**Keywords:** morusin, prenylated polyphenols, pharmafood, nutraceuticals, pro-health effect

## Abstract

Prenylflavonoids are widespread in nature. Plants are valuable sources of natural polyphenolic compounds with isoprenyl groups, which include flavones, flavanones, chalcones and aurones. They can be found in flowers, bark and stems. One of the most important compounds found in the bark of white mulberry (*Morus alba*) is morusin, a prenylated flavone with interesting pro-health properties. The research carried out so far revealed that morusin has antioxidant, antitumor, anti-inflammatory and anti-allergic activity. Moreover, its neuroprotective and antihyperglycemic properties have also been confirmed. Morusin suppresses the growth of different types of tumors, including breast cancer, glioblastoma, pancreatic cancer, hepatocarcinoma, prostate cancer, and gastric cancer. It also inhibits the inflammatory response by suppressing COX activity and iNOS expression. Moreover, an antimicrobial effect against Gram-positive bacteria was observed after treatment with morusin. The objective of this review is to summarize the current knowledge about the positive effects of morusin on human health in order to facilitate future study on the development of plant polyphenolic drugs and nutraceutics in the group of prenylflavones.

## 1. Introduction

Polyphenols belong to natural substances with interesting biological activity [1]. A promising group are natural prenylflavonoids which are characterized by a broad spectrum of pharmacological activity [2,3].

White mulberry (*Morus alba*) is a leafy tree native to Southeastern Asia. Its leaves, fruits, seeds and bark contain a range of valuable bioactive compounds, such as flavonoids (astragalin, kaempferol, quercetin, rutin) and alkaloids (1-deoxynojirimycin).

Morusin belongs to the group of flavonoids. It is one of the most important prenylated flavones naturally occurring in the root cortex of *Morus alba* (Moraceae). Morusin is a compound with the chemical formula C_25_H_24_O_6_, the molecular weight of 420.5 g/mol and the IUPAC name 2-(2,4-dihydroxyphenyl)-5-hydroxy-8,8-dimethyl-3-(3-methylbut-2-en-1-yl)-4H,8H-pyrano[2,3-f]chromen-4-one. It has the chemical structure of a flavone with hydroxyl groups at C-5, C-2′ and C-4′. These free hydroxyl groups may be substituted with various functional groups, affording structural analogues of morusin. Morusin is a hydrophobic hydroxyflavone, which is practically insoluble in water, meaning that it is also very poorly soluble in body fluids. For this reason, new methods to increase its in vivo efficacy are being developed [4].

**Figure 1:**
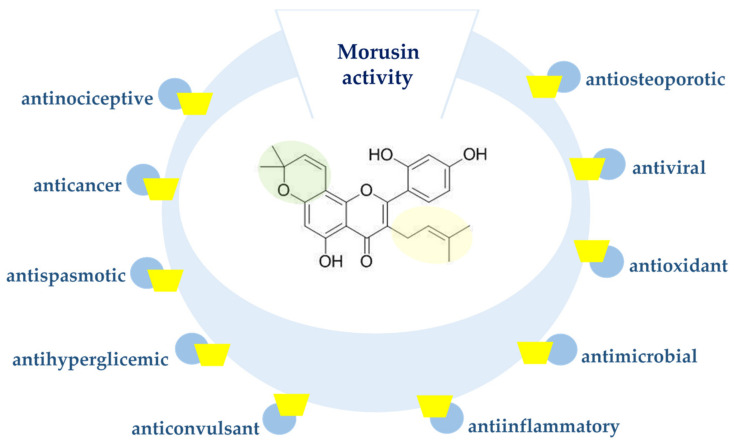
Biological activity of morusin.

Moreover, morusin was proved to have anticancer [5,6], antioxidant [7], anti-inflammatory [8] and antimicrobial [9] activity (Figure 1). This compound might also serve as a potential inhibitor of human immunodeficiency virus (HIV) [10]. Recently, morusin was also identified as an active antiviral component of *Mori ramulus* against herpes simplex virus type 1 (HSV-1) [11].

This literature review aims to systematize the current state of knowledge of the pro-health properties of morusin in order to turn the attention of researchers and medical doctors to the possibility of its use to prevent and treat certain diseases in the future.

## 2. Sources and Metabolism of Morusin

### 2.1. Morusin Identified in Plants

Various parts of plants, mainly of the family *Moraceae*, contain a range of phenolic substances, also including prenylated flavonoids (though in smaller amounts). Predominantly, they contain free isoprenyl chains, but some of them underwent earlier cyclisation to pyran or furan ring systems with diverse chemical structures.

Morusin is an aglycone, a derivative of hydrogenated flavone which has no double bond between C-2 and C-3. In nature, rich sources of morusin are plants such as *Morus alba*, *Morus nigra*, *Morus notabilis*, *Morus lhou*, *Morus australis*, *Artocarpus tonkinensis*, *Artocarpus altilis* and *Ramulus mori* (Table 1).

### 2.2. Chemical Synthesis of Morusin

Chemical synthesis is regarded as an effective and fast method of obtaining pure and biologically active substances that are structural analogues of naturally occurring compounds. However, in the case of morusin and other prenylated flavonoids, this is usually a multi-step process, difficult to perform and leading to low yields of products. An additional difficulty is the presence of the stereogenic center at C-3 in prenylflavonoids, such as in 8-prenylnaringenin, which is the most potent phytoestrogen of plant origin. A typical achiral synthesis of such compounds leads to racemic mixtures, which require further separation steps to achieve pure enantiomers.

**Figure 2:**
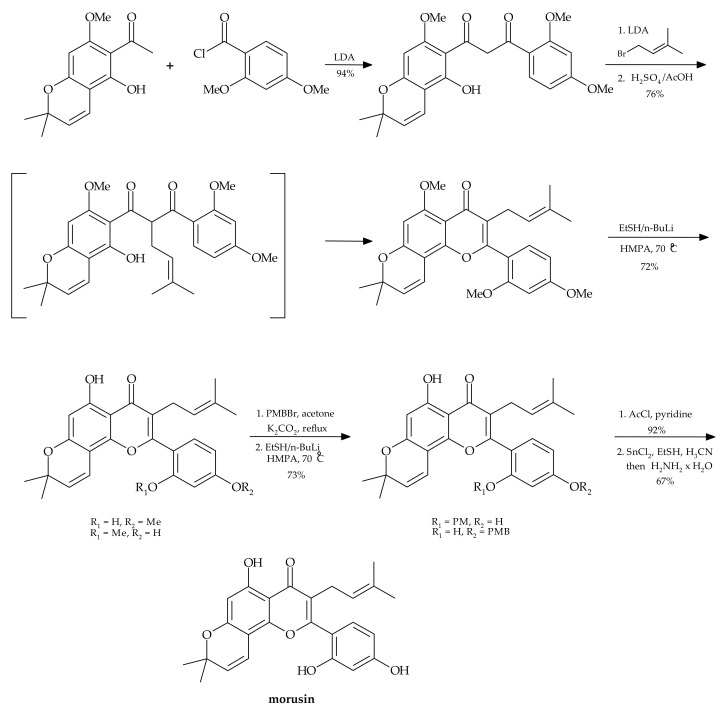
Chemical synthesis of morusin [23].

In the literature, we can find the chemical synthesis of morusin from commercially available phloroglucinol, proposed by Tseng et al. [23]. This is a 13-step process affording the target molecule in only 12% yield (Figure 2).

The same authors made an attempt to perform regioselective demethylation of polymethylated morusin using metal/EtSH reagents. However, the attempts to use such reagents as trimethylsilyl chloride (Me_3_SiI), aluminum chloride (AlCl_3_), boron tribromide (BBr_3_), BBr_3_/NaI, SiCl_4_/NaI, pyridinium hydrohalides and EtSLi for obtaining morusin were unsuccessful.

### 2.3. Metabolism of Morusin

Metabolism of flavonoids occurs in liver, with the help of cytochrome P450 enzymes. These are divided to phase I enzymes, which catalyze hydroxylation and demethylation reactions, and phase II enzymes, catalysators of *O*-methylation, coupling with glucuronic acid or sulfuric acid. The products of flavonoid metabolism are excreted with urine and bile, being a part of enterohepatic circulation, which prolong their excretion time of and the time of their possible activity. The metabolites of flavonoids unabsorbed and secreted with bile are transformed by intestinal microflora, which takes place mainly in the large intestine. Bacterial enzymes are able to catalyze reactions such as hydrolysis of glucuronides, sulphates and glycosides, dihydroxylation, demethylation, double bond reduction, cleavage of the C-ring along with formation of phenolic acids, followed by their decarboxylation. So far, reports on pharmacokinetic studies involving morusin are scarce [24]. Moreover, there is no information about transformations of morusin by microbial cells, including yeasts and filamentous fungi.

**Figure 3:**
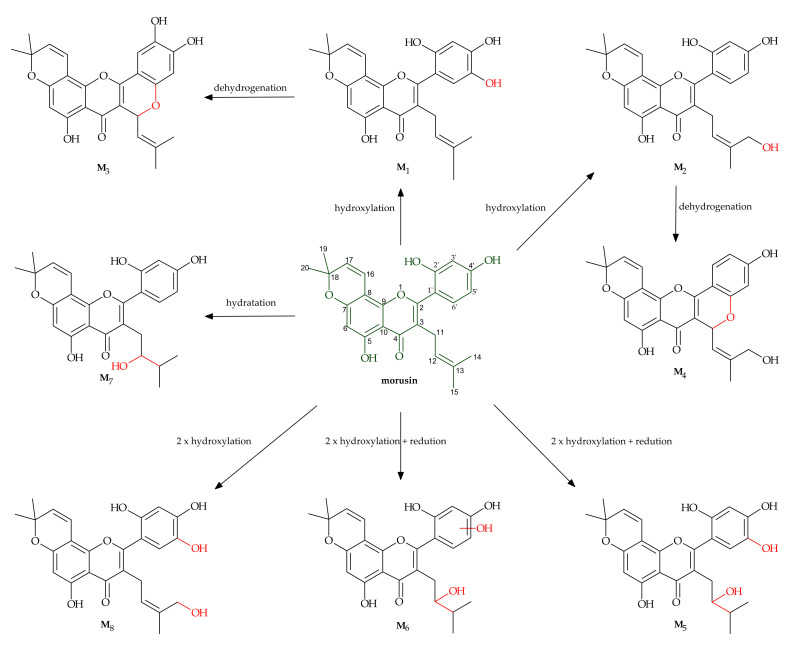
Metabolism of morusin by hepatic microsomes [25].

Shi et al. [25] described their in vitro study on the liver metabolism of morusin. The metabolites are listed in Table 2, whereas their chemical structures and proposed metabolic pathways are presented in Figure 3. The identified metabolites of morusin differed depending on the species the liver has come from. Eight metabolites were detected in the liver microsomes of pigs, rats and apes. Meanwhile, in humans, six metabolites were identified, and similarly in rabbits and dogs. In all the species, metabolites M_1_, M_2_, M_5_ and M_7_ were observed, and hydroxylation was the main process of morusin transformation. The results of the study led to the conclusion that among the cytochrome P450 enzymes, CYP3A4 that played the most important role in the transformation of the substrate by the liver.

The research showed that two metabolites of morusin, M_1_ and M_2_, were the products of hydroxylation occurring at C-5 and C-14, respectively. M_3_ and M_4_ had the same molecular formula of C_25_H_22_O_7_ and showed a 𝑚/𝑧 of 2 Da less than for M_1_ or M_2_, and for this reason, they were identified as the products of cyclization and dehydrogenation of M_1_ and M_2_. M_5_ and M_6_ were also isomers with the same molecular formula of C_25_H_26_O_8_ (𝑚/𝑧 453.1515) but had different retention times and were identified as the products of reduction and dihydroxylation of morusin. Metabolite M_7_ was identified as the product of morusin hydration with the hydroxyl group at C-12, and M_8_ as the product of dihydroxylation.

The group of Shi et al. [26] incubated morusin (1–200 µM) with human, rat, monkey, dog and minipig liver microsomes. Three products were observed when morusin (10 mM) was incubated with the hepatic microsomes from monkey and minipig (0.3 mg protein/mL) along with the NADPH-generating system for 60 min, whereas two products (P1 and P3) were observed for human, dog and rat microsomes. The quantities of one of them (P1) generated by minipig, monkey, dog and rat were 7.8-, 11.9-, 2.0- and 6.3-fold that of the human levels, respectively. It was determined that morusin at the concentration of 100 mM has an inhibiting effect on CYP450 cytochrome enzymes and UDP-dependent glucosyltransferases (UGTs). It was also proved that it belongs to competitive inhibitors of CYP1A2, CYP2E1 and UGT1A6 enzymes. Additionally, it was established that inhibition of CYP3A4, CYP2C9, UGT1A7 and UGT1A8 enzymes with the help of morusin is a non-competitive process. The above-mentioned isoforms of CYP450 play the most important role in phase I metabolism in the liver; thus, the inhibiting effect of morusin may have a significant effect on concentration of drugs administered together with morusin, which are excreted by liver metabolism.

## 3. Biological Effects of Morusin and Its Derivatives

### 3.1. Anticancer Properties

Cancers are one of the most serious diseases of modern civilization. Chemotherapy currently used to treat cancers, except for cancer cell-cycle dysregulation, affects also normal cells and causes a range of side effects, which negatively affect the overall health condition of patients. For this reason, there is wide interest in new, more efficient and safer therapeutic methods that would selectively eliminate only cancer cells. Natural compounds, including polyphenols with an isoprenyl group, due to their high potential for medical use, nowadays play an important role in the development of new molecularly targeted drugs, dietary supplements or antimicrobial agents [27]. Along with the development of biological therapeutic methods, these substances started to be used as standards or as substrates to obtain new derivatives [28].

Morusin shows in vitro anticancer activity against some types of human cancers (Table 3), such as colon, prostate, breast, cervical and hepatic one [29,30,31].

In addition, morusin suppresses the growth of breast cancer cells through C/EBPβ and PPARγ-mediated lipoapoptosis [32] and induces cell death by inactivating STAT3 signaling in prostate cancer cells [30].

The research results showed that morusin has an inhibiting effect also on the MCF-7 breast cancer cell line, depending on concentration and time of action. The established IC_50_ value in MTT assay was 3.4 μM after 24 h of exposition and 7.88 µM after 72 h of exposition. High activity of morusin was also confirmed in the study with human lung cancer cells A-549 (IC_50_ = 3.1 µM) and cervical cancer HeLa cells (IC_50_ = 0.64 µM). Moreover, morusin considerably inhibits the growth and clonogenicity of human colon carcinoma cells HT-29 [33]. Additionally, it inhibits phosphorylation of IKK-α, IKK-β and IκB-α, increases the expression of IκB-α, and suppresses nuclear translocation of NF-κB, which affects its DNA binding activity. Moreover, after administration of morusin, the authors also observed the activation of caspase-8, change in mitochondrial membrane potential, release of cytochrome c and Smac/DIABLO and activation of caspase-9 and caspase-3, which directly affect apoptosis of cancer cells.

Yin et al. [34] proved that morusin at a concentration of 10 and 30 µg/mL significantly inhibits the growth and migration of A549 cells. In addition, it increases their antioxidant potential, induces changes in mitochondrial membrane permeability and downregulates genes responsible for angiogenesis.

Morusin can also induce non-apoptotic cell death and cause autophagocytosis. One of the specific markers for autophagy is microtubule-associated light chain 3 (LC3), which is the mammalian homologue of the yeast protein Apg8p. The processed form of this protein in the in cells undergoing autophagocytosis is LC3-II.

Cho et al. [35] examined whether morusin induces autophagy in cancer cells. According to the obtained results, morusin induces LC3-II accumulation and ULK1 activation in HeLa cells. In addition, it was found that induction of ULK1 Ser317 phosphorylation and reduction of ULK1 Ser757 phosphorylation occurred simultaneously during morusin-induced autophagy. Morusin induces also autophagy by activation of AMPK and inhibition of the mTOR activity pathway.

Kang et al. [36] reported that morusin reduces the viability of human breast cancer cells (MCF-7, MDA-MB-231, MDA-MB-157 and MDA-MB-453) in a dose-dependent and time-dependent manner, while not affecting normal human breast epithelial cells (MCF10A). In addition, morusin reduces the activity of STAT3 by inhibiting its phosphorylation, nuclear accumulation and DNA-binding activity. Moreover, morusin downregulates the expression of genes encoding Bcl-xL, Bcl-2, survivin, c-Myc and cyclin D1 in the morusin-induced cells.

Gao et al. [37] found that morusin exerted growth inhibition effects on human HCC cells (HepG2 and Hep3B) in vitro and human HCC cell (HepG2) xenografts in vivo. Interleukin 6 (IL-6) is one of the main inflammatory mediators in cancer pathogenesis. In this study, morusin suppressed constitutive as well as IL-6-induced STAT3 phosphorylation in HCC cells and corresponding tumor tissues.

Park et al. [38] studied the influence of morusin on sensitization of human glioblastoma cells to apoptosis mediated by tumor necrosis factor-related apoptosis-inducing ligand (TRAIL). The activity of this ligand is associated with the expression of death receptors TRAIL-R1 (DR4) and TRAIL-R2 (DR5) in cancer cells. The authors reported that morusin induced expression of DR5, but not DR4. In addition, it reduced expression of EGFR and PDFGR, suppressed phosphorylation of STAT3 and decreased anti-apoptotic molecules. Combination treatment of TRAIL with morusin decreased cancer cell viability and increased apoptosis, compared with single treatment with either TRAIL or morusin.

The research results show that the anticancer effect of morusin is correlated with inhibition of signal transducer and activator of transcription 3 (STAT3) and/or nuclear factor NFκB, both involved in carcinogenesis. Kim et al. [39] confirmed the inhibitory activity of morusin to STAT3, which is overexpressed in pancreatic cancer cells, where it is responsible for proliferation and inhibition of apoptosis. The authors studied the effect of morusin on AsPC-1, BxPC-3, MIAPaCa-2 and PANC-1 pancreatic cancer cell lines. Morusin specifically inhibited constitutive STAT3 activation both at tyrosine residue 705 and serine residue 727 in four pancreatic tumor cells. The inhibition of STAT3 was mediated through the suppression of activation of upstream JAK1, JAK2 and c-Src kinases. It was also evidenced that the expression of several STAT3-regulated gene products was downregulated by morusin. These included antiapoptotic (Bcl-2, Bcl-xl, IAP1, IAP2 and survivin), proliferative (cyclin D1 and COX-2) and metastatic (MMP-9 and VEGF) gene products.

In another study, Lin et al. [40] evaluated the influence of morusin on the SK-Hep1 cell line (human adenocarcinoma having no properties of hepatocytes). Morusin in non-toxic concentrations suppressed cancer cell invasion, increased the expression of E-cadherin and decreased the expression of vimentin and α2-, α6- and β1-integrin. Additionally, morusin reduced the activity of metalloproteinases MMP-2 and MMP-9. Moreover, it was confirmed that morusin suppressed transcription factors STAT3 and NFκB signaling pathways of SK-Hep1 cells.

Wang et al. [41] proved that morusin suppresses the growth of human cervical cancer stem cells (CSC), which are responsible for tumor metastasis. The mechanism of its action includes inhibition of the activity of NF-ĸBp65 in cytosol and in the cell nucleus, inhibition of cell proliferation and migration, decreasing the expression of Bcl-2 protein, increasing the expression of pro-apoptotic proteins Bax and caspase-3 and induction of DNA fragmentation after 48 h treatment of the cells with morusin at the concentrations of 1.0, 2.0 and 4.0 µM. Morusin was able to upregulate the expression of both caspase-3 and caspase-9 in Bel-7402 cells [42].

Epithelial ovarian cancer (EOC) is the most common cause of death among women who develop cancers of gynecologic origin. According to Xue et al. [43], morusin inhibits proliferation and viability of EOC cells both in vitro and in vivo. The cell death occurred by cytoplasmic vacuolization due to dilation of the endoplasmic reticulum and mitochondria. Moreover, it was demonstrated that morusin induced increases in Ca^2+^ levels in mitochondria, accumulation of ER stress markers, ROS generation and loss of mitochondrial membrane potential in EOC cells.

In addition, it was noted that morusin has the ability to inhibit human glioblastoma stem cell growth in vitro and in vivo through adipocyte trans-differentiation and induction of apoptosis [44].

Furthermore, morusin protects neuronal cells from death [45] and inhibits proliferation and growth of human gastric cancer cells (MKN45, SGC7901) when treated with a dose of 2 mg/L for 72 h [46].

### 3.2. Antioxidant Activity

Polyphenols belong to the group of natural antioxidants. Their antioxidant properties arise from the presence of free hydroxyl groups in their structures. In this way, they protect an organism from harmful effects of free radicals. The activity of polyphenols is based on neutralization of reactive oxygen species, chelation of transition metals which prevents the formation of the hydroxyl radical in cells, protection of small-molecule antioxidants from oxidation and inhibition of lipoprotein oxidation.

Mulberry leaves are a rich source of antioxidants and have the capability to neutralize free radicals, being responsible for oxidative stress and thereby cell damage [12,47]. Abbas et al. [48] used the ATBS assay to demonstrate that both prenylation and cyclization of the isoprenyl group present in natural compounds from *Morus nigra*. decrease their antioxidant activity. For example, morusin, in which the prenyl group at C-8 undergoes cyclization to the pyran ring, showed lower capability to scavenge free radicals (61.34%) than its analogue kuwanon C with two free isoprenyl groups at C-5 and C-8 (80.38% activity).

Cheng et al. [49] evaluated the effect of morusin on TPA-induced ROS production. It was shown that morusin, due to its high antioxidant potential, may suppress the development of some types of cancer. Non-cytotoxic concentrations of morusin were found to dose-dependently reduce TPA-induced ROS production. By using the JB6 P^+^ cell model, it was found that morusin inhibited TPA-induced activator protein-1 (AP-1) and nuclear factor-kappa B (NF-κB) activation, which can mediate cell proliferation and malignant transformation. Furthermore, morusin inhibited the TPA upregulation of cyclooxygenase-2 (COX-2), which may be regulated by AP-1 and NF-κB. In addition, morsuin reduced the TPA-induced motility and expression of N-cadherin and vimentin, which are associated with the GSH level. In the cellular antioxidant assay (CCA) test performed by Treml et al. [50], morusin was inactive (Table 4).

### 3.3. Antibacterial and Antiprotozoal Activity

Natural prenylated flavones, flavanones and chalcones are known for their antimicrobial activity [27,52]. Similarly, morusin also demonstrates antibacterial and antifungal properties (Table 5). Wu et al. [53] reported the antimicrobial properties of morusin, mainly against Gram (+) bacteria of the genera *Bacillus*, *Enterococcus* and *Staphylococcus*.

Meanwhile, Gram (-) bacteria remained resistant to morusin. This may arise directly from the different structures of G (+) and G (-) cell membranes. Antibacterial activity of morusin towards Gram (+) bacteria was also confirmed in the study with the strains *S. epidermis* ATCC 12228 and *S. aureus,* carried out by Sohn et al. [52].

A detailed study performed by of the group of Pang [6] with the help of scanning electron microscopy (SEM) and transmission electron microscopy (TEM) revealed that morusin had a destructive effect on the integrity of the bacterial cell wall and bacterial cell membrane by affecting the expression of genes associated with the cell phosphatidic acid biosynthesis pathway. Phosphatidic acid is the main precursor in biosynthesis of phospholipids stabilizing cell membranes. Similarly, in the cells of *S. aureus* treated with morusin, a change in the fatty acid profile was observed, which may be associated with inhibition of the activity of the acyltransferases which catalyze transformation of lipids in cells. The MIC values of morusin toward *S. aureus* ATCC 6538 and *S. aureus* ATCC 25923 were both 14.9 μmol L^−1^. Thus, disruption of plasma membranes in bacterial cells may also directly affect cell viability. This is in accordance with the observation that prenylation may increase lipophilicity of flavonoids, which increases their affinity to cell membranes [54].

Boonphong et al. [22] proved that morusin isolated form the roots of *Artocarpus altilis* inhibits growth of *Plasmodium falciparum*, the protozoan parasite that causes human malaria. The measured IC_50_ value for morusin was 4.5 µM. Similar activity was found for close structural analogues of morusin, such as artocarpin (IC_50_ = 6.9 µM), cudraflavone B (IC_50_ = 5.2 µM), artonin E (IC_50_ = 6.4 µM) and artobilxanthone (IC_50_ = 6.9 µM). The newest research indicates that morusin may be used for the development of combination antimicrobial therapies. Aelenei et al. [55] confirmed that morusin increases antibiotic activity and reverses antibiotic resistance in *S. aureus* and *S. epidermidis*.

### 3.4. Anti-Inflammatory Activity

Morusin displays also anti-inflammatory activity, which molecular mechanisms include inhibition of COX and lipoxygenase (LOX) activity. Cyclooxygenase plays and essential role in in the biosynthesis of prostanoids, e.g., prostaglandins, formed in transformations of arachidonic acid. Increased synthesis of this enzyme is observed in inflammation, cancers and degenerative diseases. Apart for inhibiting the activity of COX and LOX, an important property of morusin is its effect on regulation of the nuclear factor NF-κB and inducible nitric oxide synthase (iNOS) [8].

The research by Chi et al. [56] revealed that the activity of prenylated flavonoids towards COX-1, COX-2, 5-LOX and 12-LOX enzymes strongly depended on the type of the isoprenyl substituent and its location in the molecule. Prenylated flavonoids containing lavandulyl moiety at C-8 were more active toward COX-1 than these with C-8 isoprenyl group. Morusin was found to be weak COX-1 inhibitor and showed COX-2 inhibitory activity in the test using homogenate of LPS-induced RAW 264.7 macrophage cells. The tested prenylated flavonoids were better inhibitors of 5-LOX than of 12-LOX.

According to another study, morusin was reported to inhibit purified COX-1 and 5- and 12-LOX from porcine leukocytes at the concentration range of 0.1–10 M [57]. In Chi’s study [50], morusin showed IC_50_ values of over 100 µM against COXs and LOXs. Meanwhile, an assessment of morusin influence on production of nitric oxide in the lipopolysaccharide-activated mouse macrophage RAW 264.7 cells carried out by Cheon et al. [58] revealed that this compound efficiently suppressed iNOS enzyme induction at the concentration greater than 10 µM. Similar results were described by Tseng et al. with regard to the liver protective effect of morusin [59].

Jin et al. [60] investigated the effect of morusin on skin inflammation. Morusin inhibited RANTES/CCL5 and TARC/CCL17 secretion via the suppression of STAT1 and NF-κB p65 phosphorylation in TNF-α and IFN-γ-stimulated HaCaT keratinocytes, and the release of histamine and LTC₄ by suppressing 5-LO activation in PMA and A23187-stimulated MC/9 mast cells. It may have potential antiallergic compounds in inflammatory skin diseases.

Chen et al. [61] studied the influence of morusin on the level of proinflammatory mediators in BALB/c mice with the pneumonia caused by *Mycoplasma pneumoniae*. The treatment with morusin decreased the levels of pro-inflammatory cytokines IL-6, IL-1β and tumor necrosis factor TNF-α, and increased the concentration of the anti-inflammatory cytokine interleukin IL-10. Moreover, morusin treatment inhibited the activation of Wnt/β-catenin and NF-κB signaling pathways. Furthermore, the DNA amount of *M. pneumoniae* decreased by 24.6% with the morusin dose of 20 mg/kg and by 47.6% with the morusin dose of 50 mg/kg, compared with the control group.

### 3.5. Anticonvulsant Activity

Epilepsy belongs to the group of neurological disorders which result from temporary disturbances in brain activity, associated with sudden, abnormal electrical discharge in neurons, which may have various clinical causes. According to the epidemiological data, epilepsy is the second most common neurological disease (after strokes). From a neurochemical point of view, epileptic seizures reflect an imbalance between excitatory and inhibitory transmission in the central nervous system, caused by changes in glutamatergic and GABAergic neurotransmission, abnormalities in functioning of ion channels and pumps and/or disturbed cell metabolism and energy state.

The anticonvulsant drugs used in the conventional therapy are only symptomatic relief medications. Therefore, there is a search for alternative epilepsy treatment. Flavonoids may play a modulatory role in the treatment of neurodegenerative diseases because they stabilize oxidative cellular processes in the central nervous system [62]. Prenylated neuroflavanones, such as prenylnaringenin, can also act as GABA_A_ receptor modulators [63]. Anticonvulsant activity of morusin was evaluated in vivo at concentrations of 5 and 10 mg/kg using isoniazid and maximal electroshock-induced convulsions. In the maximal electroshock seizure test, administration of morusin in a dose of 20 mg/kg (LD_50_) delayed the start of convulsions and tonic hind limb extension along with the duration of the convulsions. Additionally, it significantly reduced mortality in convulsions induced by isoniazid and electroshock. Similarly, in the rats treated with morusin at the doses of 5 and 10 mg/kg, an increased level of brain GABA was observed at both tested doses [64].

### 3.6. Application of Morusin in Memory Disorders

In traditional Chinese medicine, white mulberry (*Morus alba* L.) is used as a plant with healing properties, including as a source of neuroprotective substances. Gupta et al. [65], in their in vivo study, documented that morusin has the capability to counteract the memory loss induced by aluminum trichloride (100 mg/kg) in rats. Administration of morusin to rats with memory deficiency resulted in the reduction of AlCl_3_-induced brain acetylcholinesterase activity and oxidative stress level in rat brains [65].

According to Kim et al. [18], morusin and its derivatives with the isoprenyl group at C-3, such as cyclomorusin, neocyclomorusin and kuwanon C, proved to be non-competitive inhibitors of acetylcholinesterase, the enzyme decomposing acetylcholine into choline and acetic acid. The established IC_50_ value for morusin was 36.4 µM for inhibition of acetylcholinerase and 24.08 µM for butyrylocholinesterase. All the above-mentioned compounds showed concentration-dependent inhibitory effect. Thus, morusin and its derivatives may be important agents to combat neurodegenerative disorders, such as Alzheimer’s disease, where the activity of acetylcholinesterase (EC 3.1.1.7) is of great importance, due to its role in hydrolysis of ester bonds in neurotransmitters, including acetylcholine, which activates cholinergic neurotransmission.

Moreover, the team of Cho et al. [66] described the inhibiting action of morusin on β-secretase 1 (BACE-1, β-amyloid cleaving enzyme 1), which is a transmembrane aspartyl protease involved in Alzheimer’s disease (AD) pathogenesis. β-Secretases catalyze cleavage of the amyloid precursor protein (APP), which leads to the formation of protein deposits in the brain, associated with the aggregation of β-amyloid. The IC_50_ value of morusin for BACE-1 inhibition was 59.4 μM, whereas its structural analogue kuwanon C with two isoprenyl groups at C-3 and C-5 was almost 20 times more active (IC_50_ = 3.4 µM).

### 3.7. Anti-Hiperglycemic Action

Obesity and related comorbidities are still the problems of modern civilization. Many research results indicate that prenylated polyphenols derived from plants have anti-diabetic activity [67]. They were found to be inhibitors of advanced glycation end-products (AGEs) [68], and also non-competitive inhibitors of aldose reductase 1B1 (AKR1B1 and AKR1B10) [69].

Rao et al. [70] evaluated in vitro biological activity of standardized extracts of plants, including *Morus alba* extract containing morusin and its derivatives, in treatment of long-term diabetic complications. They assessed the inhibitory activity of the extract to aldose reductase (AR) and advanced glycation end products (AGEs) formation.

Chen et al. [71] demonstrated the α-glucosidase inhibitory activity of prenylated flavonoids contained in extracts of various parts of mulberry *Morus alba* (Cortex Mori, Ramulus Mori, Folium Mori and Fructus Mori). The most active was the extract of the root bark (Cortex Mori), where the concentration of prenylated flavonoids (kuwanon G, sanggenon C, morusin and mulberroside A) determined by HPLC was the highest, compared with other extracts that contained only trace amounts of these compounds.

Yimam et al. [72] evaluated the efficacy of the standardized plant extract UP601, composed of *M. alba*, *Y. mate* and *M. officinalis* in suppressing appetite in C57BL/6J mice treated with oral doses of 1.3 g/kg/day for 7 weeks. They observed a decrease in body weight gain and calorie intake, reduction in insulin level (75.9%), reduction in leptin level (46.8%) and a 4.2-fold increase in ghrelin level in treated mice.

### 3.8. Morusin Effect on Fat Metabolism

According to the reports by [45], morusin at the concentration of 20 µM reduced by 51% accumulation of triglycerides (TG) and suppressed by 70% the activity of cytosolic glycerol-3-phosphate dehydrogenase (GPDH) in the 3T3-L1 cells. GPDH is responsible for conversion of glycerol to TG. In addition, the IC_50_ dose for inhibition of nitric oxide production in LPS-stimulated RAW 264.7 cells for morusin was 10.6 µM.

Furthermore, the research by Guo et al. [44] revealed that morusin stimulates lipid accumulation in cancers. Treatment with morusin induced a dose-dependent increase in lipid concentration in glioblastoma stem cells (GSC) and increased levels of adipogenic proteins, including peroxisome proliferator-activated receptor PPARγ, adipsin, adipose-related gene aP2 and perilipin.

A similar effect on the accumulation of lipids was observed in breast cancer cells—both human and mouse ones. The levels of expression of two adipogenic factors (C/EBP and PPARγ) and adipogenic proteins (adipsin D and perilipin) increased in morusin-treated MCF-7 cells [32].

These results, however, did not explain the mechanism of morusin function in lipid metabolism. In order to elucidate the effect of morusin on fat metabolism, a study with the use of 3T3-L1 and primary adipocytes was performed [73]. It was proved that morusin inhibits adipogenesis by regulating the insulin receptor signaling, cell cycle and expression of adipogenic protein. It was also proved that morusin stimulates lipolysis by increasing glycerol release and enhancing expression of lipolytic proteins HSL, ATG and perilipin.

### 3.9. Antispasmodic Activity

Disorder of smooth muscles contraction in the gastrointestinal tract or respiratory system is associated with either gastrointestinal diseases or asthma. Due to acquired resistance to conventional therapies, there is a search for new therapeutic strategies based on natural substances with potential antispasmodic activity [74,75]. Zoofishan et al. [12] evaluated antispasmodic activity of ingredients of *Morus nigra*. Morusin and some of its analogues, such as kuwanon E, moracin and albanol, displayed weak relaxant activity in rat ileum and moderate activity in tracheal smooth muscles.

### 3.10. Antiviral Activity

The problems with respiratory tract infections caused by coronaviruses and the lack of effective methods of treatment of viral infections in humans inspired Thabti et al. [76] to study the antiviral activity of water and water-alcohol plant extracts of *Morus* spp. The results revealed that natural products, such as kuwanon C, which can be found, for example, in the leaves and stem bark of *Morus alba*, have antiviral activity against human coronavirus HCo-V-229E. Most of the tested extracts were also highly effective against other viruses: human poliovirus, parechovirus and echovirus.

### 3.11. Antiosteoporotic Activity

Changes in bone tissue are important for proper functioning of skeletal system during bone growth in childhood and adolescence, bone remodeling in an adult organism, healing of bone fractures and maintaining of calcium and phosphate homeostasis, which is necessary for the health of the whole organism. In pathomechanism of osteoporosis, due to the loss of control on functioning of osteoclasts, osteoblasts and other bone cells and/or communication between them, there is no longer coupling between bone resorption and bone formation processes. Increased bone resorption is observed, along with high bone turnover and a high number of remodeling sites, whereas with aging bone formation, processes become less effective. Thus, there is a search for new strategies to prevent loss of bone mass and the development of osteoporosis. The employment of natural substances with an isoprenyl group to combat osteoporosis might be an interesting idea [77,78]. Recent research has shown the function of morusin on inhibiting osteoclast differentiation and proliferation in vitro, and it was also found in in vivo studies that this compound exerts anti-osteoporosis effects [79].

Lin et al. [80] demonstrated the inhibiting effect of morusin and its structural analogues on osteoclast differentiation. The determined concentration at which the authors observed the anti-osteoporosis activity was 10^−5^ mol/L. The important structural features for enhancement of the activity of tested compounds were the presence of the isoprenyl chain at C-3 and a free hydroxyl group at C-3′. The mechanism of antiosteoporotic activity of tested compounds was based on the inhibition of TRAP enzyme activity and bone resorption in osteoclasts, and also on the induction of osteoblast proliferation in vitro.

### 3.12. Antinociceptive Activity

De Souza et al. [81] investigated in vivo effects of morusin isolated from root bark of *Morus nigra* on the antinociceptive system in mice, responsible for the control of pain transmission. The obtained results unequivocally indicate that morusin has promising pain-modulating properties. It was observed that morusin was more effective in modulation of pain perception than common analgesics, such as aspirin and paracetamol. Naloxone, one of the pain relief medicines and an opioid receptor antagonist, reversed antinociceptive activity of morusin.

### 3.13. Enzyme-Inhibiting Activity

Chaita et al. [82] studied the influence of active compounds isolated from *Morus alba* and a dozen or so plant extracts on melanogenesis—a process by which skin pigments called melanins are produced in melanocytes. Melanins are synthesized with the help of tyrosinase and are stored inside melanosomes, located in melanocytes. Tyrosinase is an oxidoreductase, which is a key enzyme in melanogenesis, where it catalyzes hydroxylation of tyrosine to amino acid L-3,4-dihydroxyphenylalanine (L-DOPA) and oxidation of L-DOPA to dopaquinone.

The role of skin pigmentation is to protect against UV irradiation, which accelerates skin aging and may lead to skin cancers. In the research by Chaita et al. [82], the authors used B16F10 melanoma cells from mouse. Among tested plant extracts, a methanolic extract from *Morus alba* demonstrated a significant inhibitory activity towards intracellular tyrosinase with the established IC_50_ value of 0.4 µg/mL. Morusin at the concentration of 300 µM reduced the enzyme by 49.9%. The most efficient tyrosinase inhibitor isolated for the first time from *Morus alba* was 2,4,3′-trihydroxydihydrostilbene (IC_50_ = 0.8 µM).

In the tyrosinase inhibitory test, morusin was inactive (IC_50_ > 100 µM). Better results were obtained for its analogs such as steppogenin (IC_50_ 0.98 ± 0.01 µM), 2,4,2′,4′-tetrahydroxychalcone (IC_50_ 0.07 ± 0.02 µM), morachalcone A (IC_50_ 0.08 ± 0.02 µM), oxyresveratrol (IC_50_ 0.10 ± 0.01 µM) and moracin M (8.00 ± 0.22 µM) [83].

### 3.14. Antinephritis Activity of Morusin

Glomerulonephritis is a common disease of the excretory system, which refers to inflammation of the glomeruli. Fukai et al. [84] investigated antinephritis activity of morusin and its analogues, e.g., glabridin. The authors measured parameters such as urinary protein excretion, total cholesterol, serum creatinine and blood urea nitrogen levels in mouse glomerular disease model (Masugi-nephritis). The potency of autoxidation (radical intensity under alkaline conditions), the effect on radical intensity of sodium ascorbate and superoxide anion radical (O_2_^− ·^) scavenging activity were tested using ESR spectroscopy. The tested compounds were administered orally at a dose of 30 mg/kg/day. ESR spectroscopy revealed that morusin increased the radical intensity of sodium ascorbate and exhibited a weak scavenging activity against superoxide anion radical. Furthermore, it decreased the urinary protein excretion, serum creatinine and blood urea nitrogen level.

## 4. New Systems for Delivery of Morusin to Cells

Easy availability of flavonoids in our everyday diet, their anticancer properties and relatively low costs of production make them an important research topic all over the world. There is a constant search for new therapeutic methods using flavonoids, in which their bioavailability is increased, for example, by improvement of their permeability across cell membranes or by increasing their water solubility. The other way of functionalization of health-promoting substances that are poorly soluble under physiological conditions is the employment of water-soluble and bioinert carriers for their transport, which, in vivo, undergo transformation into harmless byproducts and do not negatively affect an organism.

Polymeric nanoparticles play a more and more important role in the development of natural nanomedicine. Due to their biocompatibility, biodegradability and the possibility to control their release at the site of interest, they find an application as carriers of drugs and medicinal substances of natural origins.

Agrwal et al. [4] synthesized nanoparticles of PLGA, the polymer which has been approved by the FDA for the use of drug delivery because it is safe for humans, biocompatible, biodegradable and easy to modify. The authors used a nano formulation composed of chlorotoxin-conjugated, morusin-loaded PLGA nanoparticles. Chlorotoxin (CTX) is a peptide derived from scorpion venom. The formulation was used for the treatment of U87 and GI-1 human glioblastoma tumors, which are difficult to cure due to low penetration of chemotherapeutics across the blood–brain barrier. Higher drug entrapment efficiency of the conjugate by cancer cells compared with normal human neuronal cells (HCN-1A) has been observed. In addition, as a result of treatment with modified PLGA–MOR–CTX nanoparticles, enhanced caspase activity, cytoskeletal destabilization, inhibition of MMP-2 metalloproteinase activity and ROS generation in the cancer cells were observed.

Earlier research by the same team [85] aimed to increase the solubility of morusin in aqueous media. The authors developed the synthesis of a niosome system (479 nm in diameter) composed of a non-ionic surfactant (sorbitan monostearate, span 60) and cholesterol using a thin-layer evaporation technique. Nanomorusin was found to be easy dispersible in aqueous media. Moreover, extended release of morusin was observed, resulting in enhanced therapeutic efficacy in HT-29, PANC-1, MDA-MB-453, SKOV-3 and L929 cancer cell lines.

## 5. Conclusions

Morusin has research-proven interesting biological properties, among them anticancer, antibacterial, anti-inflammatory, antiallergic and antihyperglycemic ones. However, because of its hydrophobic character and thus poor water solubility, morusin is characterized by low bioavailability and fast degradation, hindering its clinical use. In addition, methabolic pathways of morusin in humans and animals have not been fully investigated so far. Similarly, synergistic activity of morusin combined with drugs or other biologically active substances from food still require more detailed analysis with respect to their health-promoting activity and effects on the human organism. Another important aspect of research on morusin is the determination of its antiviral activity.

Nevertheless, there is a great possibility to increase the use of plant raw materials in the production of nutraceutics and plant medicines. Therefore, aiming at future practical application of morusin in medicine, a multi-aspect pharmacokinetic study is needed to confirm its efficacy under physiological conditions in vivo.

The objective of this review article was to support future development of plant medicines based on prenylated flavonoids isolated from *Morus alba*, including morusin.

## Figures and Tables

**Table 1:** The natural sources of morusin.

Plant	Part of Plant	Ref.
*Morus nigra*	root bark	[12]
*Morus alba*	root bark	[13]
*Morus notabilis*	twigs	[14]
*Mori Cortex Radicis*	-	[15]
*Artocarpus tonkinensis*	roots	[16]
*Artocarpus altilis*	stem bark	[17]
*Morus lho* *u*	root bark	[18]
*Morus australis*	cortex	[19,20]
*Ramulus mori*	mulberry Husang-32	[21]
*Artocarpus altilis*	root	[22]

**Table 2:** The metabolites of morusin identified by mass spectrometry in human and animal liver microsomes [26].

Metabolite	Chemical Formula	Species					
		Rat	Monkey	Pigs	Rabbits	Human	Dog
morusin	C_25_H_24_O_6_	+	+	+	+	+	+
M_1_	C_25_H_24_O_7_	+	+	+	+	+	+
M_2_	C_25_H_24_O_7_	+	+	+	+	+	+
M_3_	C_25_H_22_O_7_	+	+	+	+	+	-
M_4_	C_25_H_22_O_7_	+	+	+	-	-	+
M_5_	C_25_H_26_O_8_	+	+	+	+	+	+
M_6_	C_25_H_26_O_8_	+	+	+	-	+	+
M_7_	C_25_H_26_O_7_	+	+	+	+	+	+
M_8_	C_25_H_24_O_8_	+	+	+	+	-	-

+ (detected); - (not detected).

**Table 3:** Anticancer activity of morusin against human cancer cell lines.

Cell Line		Test	Time	IC_50_ (µM)	Ref.
MDA-MB-231	breast adenocarcinoma	MTT	24 h	3.2	[23]
MCF-7	breast cancer			3.4	
A549	lung cancer			3.1	
DU145	prostate cancer	MTT	24 h	26.27	[30]
M2182				22.19	
PC3				19.97	
LNCaP				21.89	
RWPE-1				43.48	
HeLa	cervical cancer	MTT	72 h	0.64	[29]
Hep-3B	hepatocellular carcinoma			9.21	
MCF-7	breast cancer			7.88	

**Table 4:** Antioxidant activity of morusin.

Antioxidant Assays	Effect	Ref.
ABTS	IC_50_ (µM): 297.83	[4]
DPPH	IC_50_ (µM): 1819.83	
iron reducting power (%)	IC_50_ (µM): 13.54	
ABTS	% inhibition: 61.34	[44]
CAA	no effect	[50]
DPPH	ED_50_ (µg mL^−1^): 100.31 (30 min), 85.24 (1 h) 70.6 (3 h) 68.29 (6 h)	[51]

**Table 5:** Antimicrobial activity of morusin.

Strains	MIC (µg/mL)	Ref.
MSSA ATCC 29123	8	[53]
MRSA T144	8	
*B. subtilis* ATCC 6051	4	
*E. faecalis* VRE1010798	8	
*E. coli* ATCC 25922	>128	
*E. coli* B2	>128	
*P. aeruginosa* 14	>128	
*K. pneumoniae* WNX-1	>128	
*S. aureus ATCC6538*	6.3	[9]
*S. aureus ATCC25923*	6.3	
*Salmonella ATCC9120*	200	
*Salmonella DSM4224*	250	
*C. albicans*	>60	[52]
*S. typhimurium KCTC1926*	>100	
*S. epidermis ATCC 12228*	20	
*S. aureus*	25	

## Data Availability

All data are publicly available.

## Abbreviations

APP	amyloid precursor protein
ATBS	2,2′-azino-bis(3-ethylbenzothiazoline-6-sulfonic acid)
BACE-1	β-amyloid cleaving enzyme 1
CCA	cellular antioxidant assay
COX	cyclooxygenase
CTX	chlorotoxin
FDA	Food and Drug Administration
GABA	gamma-aminobutyric acid
GPDH	glycerol-3-phosphate dehydrogenase
GSH	glutathione
HIV	the human immunodeficiency viruses
HSV-1	herpes simplex virus 1
L-DOPA	L 3,4 dihydroxyphenylalanine
LOX	lipoxygenase
LPS	lipopolysaccharide
MIC	minimum inhibitory concentration
NFκB	nuclear factor kappa B
PLGA	poly(lactic-co-glycolic acid)
ROS	reactive oxygen species
STAT3	signal transducer and activator of transcription 3
TEM	transmission electron microscopy
TG	triglycerides
TRAIL	TNF-related apoptosis-inducing ligand
TRAP	tartrate-resistant acid phosphatase

## References

[1] Stompor-Gorący M., Bajek-Bil A., Machaczka M. (2021). Chrysin: Perspectives on contemporary status and future possibilities as pro-health agent. Nutrients.

[2] Turdo A., Glaviano A., Pepe G., Calapà F., Raimondo S., Fiori M., Carbone D., Basilicata M., Di Sarno V., Ostacolo C. (2021). Nobiletin and xanthohumol sensitize colorectal cancer stem cells to standard chemotherapy. Cancers.

[3] Koosha S., Mohamed Z., Sinniah A., Ibrahim Z.A., Seyedan A., Alshawsh M.A. (2019). Antiproliferative and apoptotic activities of 8-prenylnaringenin against human colon cancer cells. Life Sci..

[4] Agarwal S., Mohamed M.S., Mizuki T., Maekawa T., Kumar D.S. (2019). Chlorotoxin modified morusin–PLGA nanoparticles for targeted glioblastoma therapy. J. Mater. Chem. B.

[5] Park H.-J., Min T.-R., Chi G.-Y., Choi Y.-H., Park S.-H. (2018). Induction of apoptosis by morusin in human non-small cell lung cancer cells by suppression of EGFR/STAT3 activation. Biochem. Biophys. Res. Commun..

[6] Zhou Y., Li X., Ye M. (2021). Morusin inhibits the growth of human colorectal cancer HCT116-derived sphere-forming cells via the inactivation of Akt pathway. Int. J. Mol. Med..

[7] Martins B.D.A., Sande D., Solares M.D., Takahashi J.A. (2020). Antioxidant role of morusin and mulberrofuran B in ethanol extract of *Morus alba* roots. Nat. Prod. Res..

[8] Jia Y., He W., Zhang H., He L., Wang Y., Zhang T., Peng J., Sun P., Qian Y. (2020). Morusin ameliorates IL-1β-induced chondrocyte inflammation and osteoarthritis via NF-ĸB signal pathway. Drug Des Develop Ther..

[9] Pang D., Liao S., Wang W., Mu L., Li E., Shen W., Liu F., Zou Y. (2019). Destruction of the cell membrane and inhibition of cell phosphatidic acid biosynthesis in Staphylococcus aureus: An explanation for the antibacterial mechanism of morusin. Food Funct..

[10] Syahdi R.R., Mun’Im A., Suhartanto H., Yanuar A. (2012). Virtual screening of Indonesian herbal database as HIV-1 reverse transcriptase inhibitor. Bioinformation.

[11] Kim T.I., Kwon E.B., Oh Y.C., Go Y., Choi J.G. (2021). Mori rhamulus and its major component morusin inhibit herpes simplex virus type 1 replication and the virus induced reactive oxygen species. Am. J. Chin. Med..

[12] Zoofishan Z., Hohmann J., Hunyadi A. (2018). Phenolic antioxidant of Morus nigra roots, and antitumor potential of morusin. Phytochem Rev..

[13] Li M., Wu X., Wang X., Shen T., Ren D. (2017). Two novel compounds from the root bark of *Morus alba* L. Nat. Prod. Res..

[14] Zhen P., Ni G., Chen X.G., Chen R.Y., Yang H.Z., Yu D.Q. (2015). Chemical constituent form Morus notabilis and their cytotoxic effect. Yao Xue Xue Bao.

[15] Lee H.J., Lyu da H., Koo U., Nam K.W., Hong S.S., Kim K.O., Kim K.H., Lee D., Mar W. (2012). Protection of prenylated flavonoids from Mori Cortex Radicis (Moraceae) against nitric oxide-induced cell death in neuroblastoma SH-SY5Y cells. Arch Pharm Res..

[16] Ma J.-P., Qiao X., Pan S., Shen H., Zhu G.-F., Hou A.-J. (2010). New isoprenylated flavonoids and cytotoxic constituents from Artocarpus tonkinensis. J. Asian Nat. Prod. Res..

[17] Shamaun S.S., Rahmani M., Hashim N.M., Ismail H.B.M., Sukari M.A., Lian G.E.C., Go R. (2010). Prenylated flavones from Artocarpus altilis. J. Nat. Med..

[18] Kim J.Y., Lee W.S., Kim Y.S., Curtis-Long M.J., Lee B.W., Ryu Y.B., Park K.H. (2011). Isolation of cholinesterase-inhibiting flavonoids from Morus lhou. J. Agric. Food Chem..

[19] Syah Y.M., Juliawaty L.D., Achmad S.A., Hakim E.H., Takayama H., Said I.M., Latip J. (2008). Phenolic constituents from the wood of Morus australis with cytotoxic activity. Z Naturforsch C J Biosci..

[20] Ko H.-H., Wang J.-J., Lin H.-C., Wang J.-P., Lin C.-N. (1999). Chemistry and biological activities of constituents from Morus australis. Biochim. et Biophys. Acta (BBA)-Gen. Subj..

[21] Wan L.Z., Ma B., Zhang Y.Q. (2014). Preparation of morusin from Ramulus mori and its effects on mice with transplanted H22 hepatocarcinoma. Biofactors.

[22] Boonphong S., Baramee A., Kittakoop P. (2007). Antitubercular and antiplasmodial prenylated flavones from the roots of Artocarpus altilis. Chiang Mai J. Sci..

[23] Tseng T.-H., Chuang S.-K., Hu C.-C., Chang C.-F., Huang Y.-C., Lin C.-W., Lee Y.-J. (2010). The synthesis of morusin as a potent antitumor agent. Tetrahedron.

[24] Deng Z., Sun X., Yang S., Zhang L., Sun P., Teng X., Zhong H. (2017). Quantification of morusin using LC-MS in rat plasma: Application to a pharmacokinetic study. Biomed. Chromatogr..

[25] Shi X., Mackie B., Zhang G., Yang S., Song Y., Su D., Liu Y., Shan L. (2016). Identification of the metabolic enzyme involved Morusin metabolism and characterization of its metabolites by ultraperformance liquid chromatography quadrupole time-of-flight mass spectrometry (UPLC/Q-TOF-MS/MS). Evid.-Based Complement. Altern. Med..

[26] Shi X., Yang S., Zhang G., Song Y., Su D., Liu Y., Guo F., Shan L., Cai J. (2015). The different metabolism of morusin in various species and its potent inhibition against UDP-glucuronosyltransferase (UGT) and cytochrome p450 (CYP450) enzymes. Xenobiotica.

[27] Stompor M., Żarowska B. (2016). Antimicrobial activity of xanthohumol and its selected structural analogues. Molecules.

[28] Stompor M., Świtalska M., Wietrzyk J. (2019). The influence of a single and double biotinylation of xanthohumol on its anticancer activity. Acta Biochim. Pol..

[29] Dat N.T., Binh P.T.X., Quynh L.T.P., Van Minh C., Huong H.T., Lee J.J. (2010). Cytotoxic prenylated flavonoids from *Morus alba*. Fitoterapia.

[30] Lim S.-L., Park S.-Y., Kang S., Park D., Kim S.-H., Um J.-Y., Jang H.-J., Lee J.-H., Jeong C.-H., Jang J.-H. (2014). Morusin induces cell death through inactivating STAT3 signaling in prostate cancer cells. Am. J. Cancer Res..

[31] Park Y.-J., Choi D.W., Cho S.W., Han J., Yang S., Choi C.Y. (2020). Stress granule formation attenuates RACK1-mediated apoptotic cell death induced by Morusin. Int. J. Mol. Sci..

[32] Li H., Wang Q., Dong L., Liu C., Sun Z., Gao L., Wang X. (2015). Morusin suppresses breast cancer cell growth in vitro and in vivo through C/EBPβ and PPARγ mediated lipoapoptosis. J. Exp. Clin. Cancer Res..

[33] Lee J.C., Won S.J., Chao C.L., Wu F.L., Liu H.S., Ling P., Lin C.N., Su C.L. (2008). Morusin induces apoptosis and suppresses NF-kappaB activity in human colorectal cancer HT-29 cells. Biochem. Biophys. Res. Commun..

[34] Yin X., Lv Y., Wang S., Zhang Y.-Q. (2018). Morusin suppresses A549 cell migration and induces cell apoptosis by downregulating the expression of COX-2 and VEGF genes. Oncol. Rep..

[35] Cho S.W., Na W., Choi M., Kang S.J., Lee S.-G., Choi C.Y. (2017). Autophagy inhibits cell death induced by the anti-cancer drug morusin. Am. J. Cancer Res..

[36] Kang S., Kim E.-O., Kim S.-H., Lee J., Ahn K.S., Yun M., Lee S.-G. (2017). Morusin induces apoptosis by regulating expression of Bax and Survivin in human breast cancer cells. Oncol. Lett..

[37] Gao L., Wang L., Sun Z., Li H., Wang Q., Yi C., Wang X. (2017). Morusin shows potent antitumor activity for human hepatocellular carcinoma in vitro and in vivo through apoptosis induction and angiogenesis inhibition. Drug Des. Dev. Ther..

[38] Park D., Ha I.J., Park S.-Y., Choi M., Lim S.-L., Kim S.-H., Lee J.-H., Ahn K.S., Yun M., Lee S.-G. (2016). Morusin induces TRAIL sensitization by regulating EGFR and DR5 in human glioblastoma Cells. J. Nat. Prod..

[39] Kim C., Kim J.H., Oh E.Y., Nam D., Lee S.G., Lee J., Kim S.-H., Shim B.S., Ahn K.S. (2016). Blockage of STAT3 signaling pathway by morusin induces apoptosis and inhibits invasion in human pancreatic tumor cells. Pancreas.

[40] Lin W.L., Lai D.Y., Lee Y.J., Chen N.F., Tseng T.H. (2015). Antitumor progression potential of morusin suppressing STAT3 and NFĸB in human hepatoma SK-Hep1 cells. Toxicol. Lett..

[41] Wang L., Guo H., Yang L., Dong L., Lin C., Zhang J., Lin P., Wang X. (2013). Morusin inhibits human cervical cancer stem cell growth and migration through attenuation of NF-κB activity and apoptosis induction. Mol. Cell Biochem..

[42] Ding B., Lv Y., Zhang Y.-Q. (2016). Anti-tumor effect of morusin from the branch bark of cultivated mulberry in Bel-7402 cells via the MAPK pathway. RSC Adv..

[43] Xue J., Li R., Zhao X., Ma C., Lv X., Liu L., Liu P. (2018). Morusin induces paraptosis-like cell death through mitochondrial calcium overload and dysfunction in epithelial ovarian cancer. Chem. Interact..

[44] Guo H., Liu C., Yang L., Dong L., Wang L., Wang Q., Li H., Zhang J., Lin P., Wang X. (2014). Morusin inhibits glioblastoma stem cell growth in vitro and in vivo through stemness attenuation, adipocyte transdifferentiation, and apoptosis induction. Mol. Carcinog..

[45] Yang Z.G., Matsuzaki K., Takamatsu S., Kitanaka S. (2011). Inhibitory effects of constituents from *Morus alba* var. multicaulis on differentiation of 3T30L1 cells and nitric oxide production in RAW264.7 cells. Molecules.

[46] Wang F., Zhang D., Mao J., Ke X.-X., Zhang R., Yin C., Gao N., Cui H. (2017). Morusin inhibits cell proliferation and tumor growth by down-regulating c-Myc in human gastric cancer. Oncotarget.

[47] You S., Kim G.-H. (2019). Protective effect of Mori Cortex radicis extract against high glucose-induced oxidative stress in PC12 cells. Biosci. Biotechnol. Biochem..

[48] Abbas G.M., Fatma M., Bar A., Baraka H.N., Gohar A., Lahloub M.F. (2014). A new antioxidant stilbene and other constituents form the stem bark of *Morus nigra* L. Nat. Prod. Res..

[49] Cheng P.-S., Hu C.-C., Wang C.-J., Lee Y.-J., Chung W.-C., Tseng T.-H. (2017). Involvement of the antioxidative property of morusin in blocking phorbol ester–induced malignant transformation of JB6 P+ mouse epidermal cells. Chem. Interact..

[50] Treml J., Večeřová P., Herczogová P., Šmejkal K. (2021). Direct and indirect antioxidant effects of selected plant phenolics in cell-based assays. Molecules.

[51] Mazimba O., Majinda R.R.T., Motlhanka D. (2011). Antioxidant and antibacterial constituents from Morus nigra. Afr. J. Pharm. Pharmacol..

[52] Sohn H.-Y., Son K., Kwon C.-S., Kang S. (2004). Antimicrobial and cytotoxic activity of 18 prenylated flavonoids isolated from medicinal plants: *Morus alba* L., Morus mongolica Schneider, *Broussnetia papyrifera* (L.) Vent, Sophora flavescens Ait and Echinosophora koreensis Nakai. Phytomedicine.

[53] Wu S.C., Han F., Song M.R., Chen S., Li Q., Zhang Q., Zhu K., Shen J.Z. (2019). Natural flavones from *Morus alba* against methicillin-resistant Staphylococcus aureus via targeting the proton motive force and membrane permeability. J. Agric. Food Chem..

[54] Yang X., Jiang Y., Yang J., He J., Sun J., Chen F., Zhang M., Yang B. (2015). Prenylated flavonoids, promising nutraceuticals with impressive biological activities. Trends Food Sci. Technol..

[55] Aelenei P., Rimbu C.M., Horhogea C.E., Lobiuc A., Neagu A.-N., Dunca S.I., Motrescu I., Dimitriu G., Aprotosoaie A.C., Miron A. (2020). Prenylated phenolics as promising candidates for combination antibacterial therapy: Morusin and kuwanon G. Saudi Pharm. J..

[56] Chi Y.S., Jong H.G., Son K.H., Chang H.W., Kang S.S., Kim H.P. (2001). Effects of naturally occurring prenylated flavonoids on enzymes metabolizing arachidonic acid: Cyclooxygenases and lipoxygenases. Biochem. Pharmacol..

[57] Reddy G.R., Ueda N., Hada T., Sackeyfio A.C., Yamamoto S., Hano Y., Aida M., Nomura T. (1991). A prenylflavone, artonin E, as arachidonate 5-lipoxygenase inhibitor. Biochem. Pharmacol..

[58] Cheon B.S., Kim Y.H., Son K.S., Chang H.W., Kang S.S., Kim H.P. (2000). Effects of prenylated flavonoids and biflavonoids on lipopolysaccharide-induced nitric oxide production from the mouse macrophage cell line RAW 264.7. Planta Med..

[59] Tseng T.H., Lin W.L., Zhang C.K., Lee K.C., Tung S.Y., Kuo H.C. (2018). Protective effects of Morus Root extract (MRE) against lipopolysaccharide activated RAW264.7 cells and CCl4-induced mouse hepatic damage. Cell Physiol. Biochem..

[60] Jin S.E., Ha H., Shin H.-K., Seo C.-S. (2019). Anti-allergic and anti-inflammatory effects of kuwanon G and morusin on MC/9 mast cells and HaCaT keratinocytes. Molecules.

[61] Chen C., Wang J., Chen J., Zhou L., Wang H., Chen J., Xu Z., Zhu S., Liu W., Yu R. (2019). Morusin alleviates mycoplasma pneumonia via the inhibition of Wnt/β-catenin and NF-κB signaling. Biosci. Rep..

[62] Pinto C., Cestero J.J., Rodriguez-Galdón B., Macias P. (2014). Xanthohumol, a prenylated flavonoid from hops (*Humulus lupulus* L.) protects rat tissues against oxidative damage after acute ethanol administration. Toxicol. Rep..

[63] Benkherouf A.Y., Soini S.L., Stompor M., Uusi-Oukari M. (2019). Positive allosteric modulation of native and recombinant GABA_A_ receptors by hops prenylflavonoids. Eur. J. Pharmacol..

[64] Gupta G., Dua K., Kazmi I., Anwar F. (2014). Anticonvulsant activity of morusin isolated from *Morus alba*: Modulation of GABA receptor. Biomed. Aging Pathol..

[65] Gupta G., Chellappan D.K., Agarwal M., Ashwathanarayana M., Nammi S., Pabreja K., Dua K. (2017). Pharmacological eveluation of the recuperative effect of morusin against aluminium trichloride (AlCl3)-induced memory impairment in rats. Cent. Nerv. Syst. Agents Med. Chem..

[66] Cho J.K., Ryu Y.B., Curtis-Long M.J., Kim J.Y., Kim D., Lee S., Lee W.S., Park K.H. (2011). Inhibition and structural reliability of prenylated flavones from the stem bark of Morus lhou on β-secretase (BACE-1). Bioorganic Med. Chem. Lett..

[67] Milella L., Milazzo S., Se Leo M., Vera Saltos M.B., Faraone I., Tuccinardi T., Lapillo M., De Tommasi N., Braca A. (2014). α-Glucosidase and α-amylase inhibitors from Arcytophyllum thymifolium. J. Nat. Prod..

[68] Nakashima K., Miyashita H., Yoshimitsu H., Fujiwara Y., Nagai R., Ikeda T. (2016). Two new prenylflavonois from Epimedii herba and their inhibitory effects on advanced glycation end-products. J. Nat. Med..

[69] Seliger J.M., Misuri L., Maser E., Hintzpeter J. (2018). The hop-derived compounds xanthohumol, isoxanthohumol and 8-prenylnaringenin are toght-binding inhibitors of human aldo-keto reductases 1B1 and 1B10. J. Enzym. Inhib. Med. Chem..

[70] Rao A.R., Shyam P., Veeresham C., Asres K. (2015). Aldose reductase inhibitory and antiglycation activities of four medicinal plant standarized extracts and their main constituents for the prevention of diabetic complications. Ethiop. Pharm. J..

[71] Chen Z., Du X., Yang Y., Cui X., Zhang Z., Li Y. (2018). Comparative study of chemical composition and active components against α -glucosidase of various medicinal parts of *Morus alba* L. Biomed. Chromatogr..

[72] Yimam M., Jiao P., Hong M., Brownell L., Lee Y.C., Hyun E.J., Kim H.J., Kim T.W., Nam J.B., Kim M.R. (2016). Appetite suppression and antiobesity effect of a botanical composition composed of *Morus alba*, Yerba mate, and Magnolia officinalis. J. Obes..

[73] Lee M.R., Kim J.E., Choi J.Y., Park J.J., Kim H.R., Song B.R., Park J.W., Kang M.J., Choi Y.W., Kim K.M. (2018). Morusin functions as a lipogenesis inhibitor as well as a lipolysis stimulator in differentiated 3T3-L1 and primary adipocytes. Molecules.

[74] Frölich S., Schubert C., Bienzle U., Jenett-Siems K. (2005). In vitro antiplasmodial activity of prenylated chalcone derivatives of hops (*Humulus lupulus*) and their interaction with haemin. J. Antimicrob. Chemother..

[75] Mendel M., Chłopecka M., Dziekan N., Karlik W. (2016). Antispasmodic effect of selected citrus flavonoids on rat isolated jejunum specimens. Eur. J. Pharmacol..

[76] Thabti I., Albert Q., Philippot S., Dupire F., Westerhuis B., Fontanay S., Risler A., Kassab T., Elfalleh W., Aferchichi A. (2020). Advances on Antiviral Activity of *Morus* spp. Plant Extracts: Human coronavirus and virus-related respiratory tract infections in the spotlight. Molecules.

[77] Li J., Zeng L., Xie J., Yue Z., Deng H., Ma X., Zheng C., Wu X., Luo J., Liu M. (2015). Inhibition of osteoclastogenesis and bone resorption in vitro and in vivo by a prenylflavonoid xanthohumol from hops. Sci. Rep..

[78] Lim R., Li L., Yong E., Chew N. (2018). STAT-3 regulation of CXCR4 is necessary for the prenylflavonoid icaritin to enhance mesenchymal stem cell proliferation, migration and osteogenic differentiation. Biochim. et Biophys. Acta (BBA)-Gen. Subj..

[79] Chen M., Han H., Zhou S., Wen Y., Chen L. (2021). Morusin induces osteogenic differentiation of bone marrow mesenchymal stem cells by canonical Wnt/β-catenin pathway and prevents bone loss in an ovariectomized rat model. Stem Cell Res. Ther..

[80] Lin B., Huang J.-F., Liu X.-W., Ma X.-T., Lu Y., Zhou Y., Guo F.-M., Feng T.-T. (2017). Rapid, microwave-accelerated synthesis and anti-osteoporosis activities evaluation of morusin scaffolds and morusignin L scaffolds. Bioorganic Med. Chem. Lett..

[81] de Souza M.M., Bittar M., Cechinel-Filho V., Yunes R.A., Messana I., Monache F.D., Ferrari F. (2000). Antinociceptive properties of morusin, a prenylflavonoid isolated from Morus nigra root bark. Z Naturforsch. C J. Biosci..

[82] Chaita E., Lambrinidis G., Cheimonidi C., Agalou A., Beis D., Trougakos I., Mikros E., Skaltsounis A.-L., Aligiannis N. (2017). Anti-melanogenic properties of Greek plants. A novel depigmenting agent from *Morus alba* wood. Molecules.

[83] Zhang L., Tao G., Chen J., Zheng Z.-P. (2016). Characterization of a new flavone and tyrosinase inhibition constituents from the twigs of *Morus alba* L. Molecules.

[84] Fukai T., Satoh K., Nomura T., Sakagami H. (2003). Antinephritis and radical scavenging activity of prenylflavonoids. Fitoterapia.

[85] Agarwal S., Mohamed M.S., Raveendran S., Rochani A.K., Maekawa T., Kumar D.S. (2018). Formulation, characterization and evaluation of morusin loaded niosomes for potentiation of anticancer therapy. RSC Adv..

